# Follicular Bronchiolitis in a Nigerian Female Child: A Case Report and Review of the Literature

**DOI:** 10.1155/2016/1096953

**Published:** 2016-05-26

**Authors:** Nzechukwu Zimudo Ikeri, Godwin O. Umerah, Christopher Emeka Ugwu, Olugbenga Olusoji, Adekunle Adeyomoye, Ekanem Ekure, Adetola Olubunmi Daramola

**Affiliations:** ^1^Department of Anatomic and Molecular Pathology, Lagos University Teaching Hospital, PMB 12003, Idi-Araba, Lagos, Nigeria; ^2^Department of Anatomical Pathology, Federal Medical Centre, PMB 7001, Umuahia, Abia, Nigeria; ^3^Department of Paediatrics, Lagos University Teaching Hospital, PMB 12003, Idi-Araba, Lagos, Nigeria; ^4^Department of Surgery, University of Lagos College of Medicine and Lagos University Teaching Hospital, PMB 12003, Idi-Araba, Lagos, Nigeria; ^5^Department of Radiodiagnosis, University of Lagos College of Medicine and Lagos University Teaching Hospital, PMB 12003, Idi-Araba, Lagos, Nigeria; ^6^Department of Anatomic and Molecular Pathology, University of Lagos College of Medicine and Lagos University Teaching Hospital, PMB 12003, Idi-Araba, Lagos, Nigeria

## Abstract

Small airways diseases are not uncommon in childhood. They account for about 28.4% of hospital admissions for lower respiratory tract infections in South West Nigeria, most of which are due to respiratory syncytial virus (RSV) infection. Noninfectious causes of small airways diseases, on the other hand, are poorly recognized and rarely feature in the differential diagnoses of chronic/recurrent lower respiratory tract disease in our environment. We present a case of follicular bronchiolitis in a 2.5-year-old Nigerian female who had left upper lobectomy on account of recurrent cough and progressive shortness of breath.

## 1. Introduction

The small airways are defined as airways that have an intraluminal diameter < 2 mm [1]. Composed of the membranous, terminal, and respiratory bronchioles, they have been called the “silent zone of the lungs” due to the poor recognition of their pathophysiologic roles in lung diseases and “the lung's Achilles heel” because they are prone to collapse during expiration (especially when diseased) due to the absence of cartilage within their walls [1, 2]. Though most cases of small airways diseases are due to viral infections in children or are smoking related in adults, an increasing variety of causative factors have been recognized including collagen vascular diseases, primary immunodeficiency syndromes, hypersensitivity reactions, bone and lung transplantation, drugs, and toxins [2, 3]. In our environment, where pulmonary tuberculosis and bronchial asthma account for the majority of cases of chronic or recurrent lower respiratory tract disease, small airways diseases rarely feature in the differential diagnoses. With the aim of raising the index of suspicion for these poorly recognized entities, we report a case of follicular bronchiolitis in a 2.5-year-old Nigerian female who had upper right lobectomy on account of persistent cough and progressive shortness of breath.

## 2. Case Report

A nine-month-old female presented with one-week history of cough, catarrh, and fever at a referral hospital where she was admitted and treated for pneumonia. On discharge after two weeks of treatment, signs of respiratory distress persisted. Recurrent and worsening dyspnea was observed over a two-month period with repeated admissions. When an ordered chest X-ray showed left upper lobe consolidation and collapse, patient was started on anti-Koch's therapy. Mantoux and erythrocyte sedimentation rate test results were 5 mm and 65 mm/hr, respectively, while tests for HIV I and II were negative. She had no history of contact with anyone with chronic cough or tuberculosis and vaccinations were up to date. There was no family history of asthma or atopy, but history of poor weight gain was present. Echocardiography ordered on account of persistent dyspnea showed a small secundum atrial septal defect which subsequently closed after 6 months.

**Figure 1:**
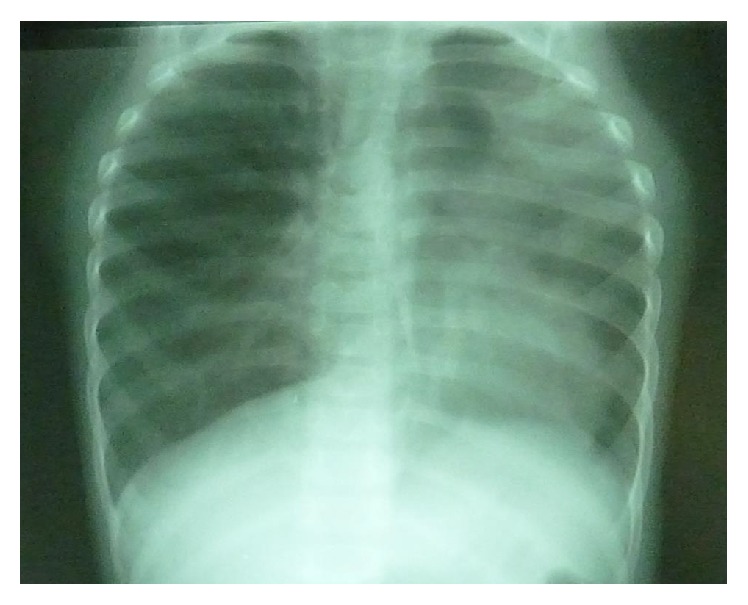
Chest X-ray showing left upper lobe collapse and mediastinal shift to the ipsilateral side.

Following 12 months of anti-Koch's therapy, an evaluation at the referral hospital revealed respiratory distress and persistent poor weight gain with weight of 8.5 kg at two years. Chest auscultation revealed widespread rhonchi and crepitations. There was no cyanosis and a repeat chest X-ray showed a hyperinflated right lung field with left upper lobe collapse and left mediastinal shift (Figure 1).

**Figure 2:**
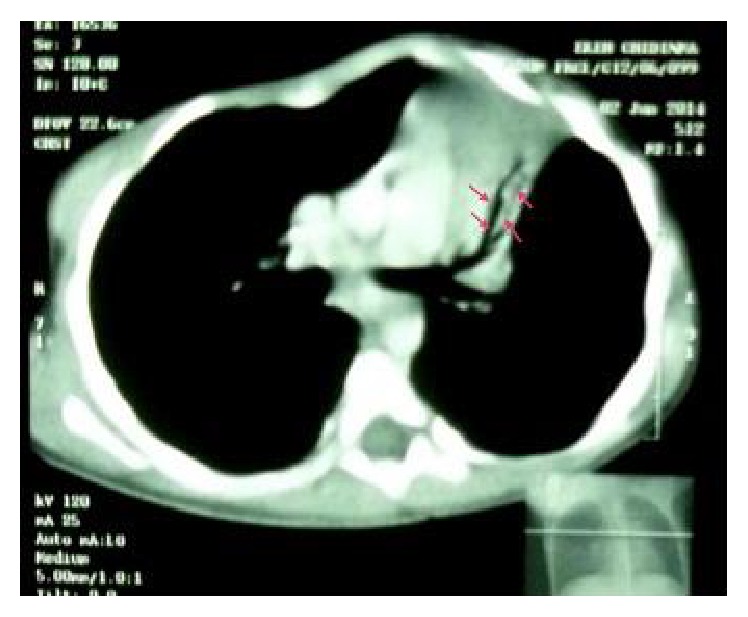
Chest CT scan showing collapse consolidation of upper left lobe with prominent air bronchograms (arrow). Note compensatory hyperinflation of the right upper lobe with shift of the mediastinum to the ipsilateral side.

**Figure 3:**
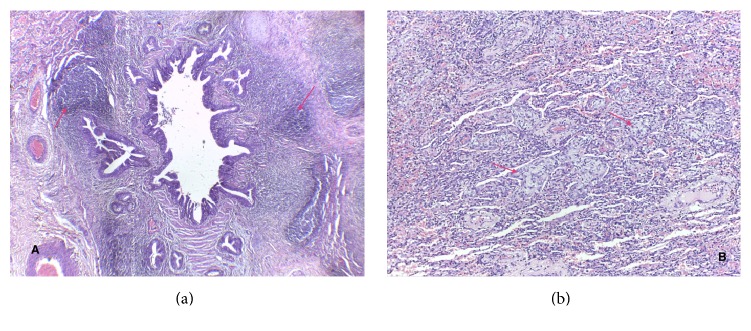
(a) Follicular bronchiolitis: hyperplastic lymphoid follicles with pale germinal centres (arrows) adjacent to a small bronchi and bronchioles (H&E ×40). (b) Organizing pneumonia: plugs of granulation tissue (Masson bodies) (arrows) within the alveolar ducts and spaces (H&E ×100).

Patient was referred to our hospital for further management. The anti-Koch's therapy was discontinued and other diagnoses such as bronchial asthma, foreign body aspiration, or congenital lung malformation were entertained. The cardiothoracic surgical unit (CTSU) was involved in the management and a requested chest CT scan showed collapse consolidation of the left upper lobe with extensive air bronchogram and compensatory hyperinflation of the right upper lobe (Figure 2). A decision to do a lobectomy was taken by the CTSU, and, at 2 years of age, she underwent left posterolateral thoracotomy and left upper lobectomy. Findings at surgery were a collapsed nodular left upper lobe, left hilar and perihilar lymphadenopathy, and healthy-looking hyperinflated left lower lobe. Histology revealed dense inflammatory infiltrates composed of lymphocytes, plasma cells, and few eosinophils within the bronchiolar walls, peribronchiolar soft tissue, and adjoining interstitium. The lymphoid cells were seen forming lymphoid follicles with pale germinal centres predominantly around the bronchi and bronchioles. Some of the bronchioles contained inflammatory debris, and plugs of granulation tissue composed of fibroblasts within a myxoid stroma were seen within the bronchiolar lumens, alveolar ducts, and associated alveolar spaces (Figure 3). Areas of haemorrhage were also seen. A diagnosis of follicular bronchiolitis was made. Postoperative period was uneventful and she was discharged home with no complications. She remains symptom-free a year after the surgery and currently weighs 12 kg at 3 years.

## 3. Discussion

Small airways diseases have become an important focus of study in the recent years, especially those cases not secondary to asthma or related to smoking [3]. Their causes are diverse and range in airway infections, connective tissue disorders, immunodeficiency syndromes, and bone marrow and lung transplantation; however, a considerable proportion of cases are idiopathic [2]. Various radiologic and pathologic classification schemes have been proposed to characterize these diseases; however, none satisfactorily covers the full spectrum of aetiologic factors [1]. To complicate things further, each of the various morphologic patterns has different clinical associations; and patients with the same clinical cause often have different morphologic forms [3]. The evaluation of each case therefore requires the integration of radiologic and pathologic findings within the proper clinical context (Table 1).

The terminologies describing bronchiolar disorders have been used in confusing fashion by both clinicians and pathologists, and though they occur quite frequently, there is no universal agreement on their classification [4]. It is therefore important to know the context in which these terms are used: whether clinical or strictly pathological [5]. In the classification scheme used here [6], cellular bronchiolitis lumps only acute bronchiolitis and acute and chronic bronchiolitis together as one, since they represent similar clinicopathological entities. The others, though cellular in a sense, have distinct clinical and radiological features (Table 1).

Follicular bronchiolitis arises as a result of altered immune response of the bronchial associated lymphoid tissue to an immune stimulus. In addition to the causes listed in Table 1, it has been reported in* Legionella pneumophila* infection, prolonged exposure to polyethylene-flock, primary ciliary dyskinesia, and multicentric Castleman disease [7–10]. Adults are more commonly affected, but it also occurs in children, where its cause is usually unknown [11, 12]. Treatment involves use of bronchodilators and steroids for cases with no known underlying cause and immune suppressive agents such as azathioprine for steroid dependent patients [12]. Kinane et al. reported a series of five cases of paediatric idiopathic follicular bronchiolitis, all of which were tachypnoeic and had chronic cough beginning at 6 weeks of age. Though response to steroids was minimal, all the patients improved at about 2–4 years of age [13]. The prognoses in these cases were much better than those presented by Yousem et al., who were all of older age groups (age range 1.5–77 years) and whose cases were secondary to autoimmune disorders, immunodeficiency states, or hypersensitivity reactions [14]. It is therefore thought that follicular bronchiolitis presenting in infancy represents a subset of the disease with a more favourable outcome [3]. This however appeared not to be the case of our patient whose disease was so severe as to necessitate surgical intervention, though she presented at nine months of age but much later than the favourable group reported by Kinane et al.

In addition to the primary forms of bronchiolitis discussed above, small airways diseases could be secondarily involved in diseases of the larger airways or by the interstitial pneumonias. Bronchiolar involvement can therefore be seen in chronic obstructive pulmonary disease (COPD) and asthma, bronchiectasis as well as lymphocytic interstitial pneumonia (LIP), usual interstitial pneumonia (UIP), and nonspecific interstitial pneumonia (NSIP) [15–18]. The interstitial pneumonias are rarely diagnosed in our institution, as open lung biopsies were not, until recently, frequently performed. However, Daramola et al. reported two cases of UIP and a case of DIP over a ten-year period from autopsy and surgical biopsy specimen [19]. Intraluminal polyps seen in the index case are the characteristic feature of organizing pneumonia with intraluminal polyps (previously called bronchiolitis obliterans organizing pneumonia; BOOP). Though included in some classification schemes of small airways disease, it is not a specific diagnosis but a descriptive histologic term for a nonspecific organizing tissue response to intraluminal exudates [3, 15, 20]. Therefore, any condition, follicular bronchiolitis, for example, that produces exudates within the lumen of the bronchioles or alveoli can potentially have an organizing pneumonia component. The presence of organizing pneumonia in follicular bronchiolitis does not portend a worse prognosis as it typically resolves with little or no scarring; however, its course and prognosis depend on the type and severity of the underlying disease [2].

Small airways diseases, as an entity, must therefore be suspected in patients presenting with chronic or recurrent lower respiratory tract symptoms, especially those refractory to antibiotic treatment. They can often be recognized in the proper clinical and high-resolution computer tomography (HR-CT) context, and as such, many cases do not require lung biopsy [4]. In the select cases where biopsy is required, an open surgical biopsy, as opposed to bronchoscopic transbronchial biopsy, is preferred, as it allows for examination of a sufficient amount of small airways [1]. Early recognition of these entities will prevent surgical measures like lobectomies that were performed in the index case.

## 4. Conclusion

Small airways diseases are potentially manageable respiratory conditions that must be suspected in patients with recurrent or chronic chest symptoms. An integration of clinical, HR-CT, and pathologic findings will often lead to the right diagnosis and allow for prompt institution of appropriate therapy.

## Figures and Tables

**Table 1:** Clinical, radiological, and pathological findings in the common small airways diseases [1, 3, 4, 6, 11, 15].

Histologic classification [6]	Clinical features	High resolution CT scan findings	Common causes
Cellular bronchiolitis	Mild dyspnea ± cough in adults; acute onset in infants; obstructive and/or restrictive pattern; good prognosis	Linear opacities or small centrilobular nodules	Infection, collagen vascular diseases, immune disorders
Nonspecific chronic bronchiolitis	Obstructive and/or restrictive pattern; variable prognosis	Linear opacities or centrilobular nodules	Infection, collage vascular diseases, posttransplantation graft versus host disease, IBD
Follicular bronchiolitis	Progressive dyspnea, chronic cough, recurrent URTI; obstructive and/or restrictive; generally good prognosis	Peribronchial nodules ± ground-glass opacities	RA, Sjogren syndrome, CVID, AIDS, hypersensitivity pneumonitis
Diffuse panbronchiolitis	Chronic productive cough, dyspnea, sinusitis; progressive airflow obstruction	Tree-in-bud appearance and centrilobular nodules	Idiopathic
Constrictive bronchiolitis obliterans	Chronic cough, dyspnea, wheeze; irreversible airflow obstruction on pulmonary function tests	Tree-in-bud pattern; low attenuation/mosaic perfusion	Lung transplant rejection, mineral dust disease, toxin/fume exposure, IBD, collagen vascular diseases
Respiratory (smoker's) bronchiolitis	Usually asymptomatic/incidental; excellent prognosis	Normal, ground-glass opacities and micronodules	Heavy smoking

IBD, inflammatory bowel disease; RA, rheumatoid arthritis; CVID, common variable immunodeficiency syndrome; AIDS, acquired immunodeficiency syndrome.
